# Fintech-driven sport event sponsorship: how commercial banks’ innovative marketing strategies shape destination branding and inclusive tourism

**DOI:** 10.3389/fspor.2026.1823565

**Published:** 2026-06-16

**Authors:** Toyirova Sarvinoz Atoevna, Qodirov Azizjon Anvarovich, Abdullayev Mehriddin Junaydulloyevich, Rajabova Dilbar Ixtiyor Qizi, Sherov Anvar Umarovich

**Affiliations:** 1 Department of Tourism and Hotel Management, Bukhara State University, Bukhara, Uzbekistan; 2 Department of Accounting and Statistics, Bukhara State University, Bukhara, Uzbekistan; 3 Department of Sport Theory and Methodology, Bukhara State University, Bukhara, Uzbekistan; 4 Department of Economics, Bukhara State University, Bukhara, Uzbekistan

**Keywords:** commercial banks, destination branding, digital payment systems, emerging economies, financial inclusion, fintech, inclusive tourism, service-dominant logic

## Abstract

The convergence of financial technology (fintech), sport event sponsorship, and destination marketing constitutes an underexplored but increasingly consequential research paradigm in tourism studies. This paper presents a conceptual analysis of how commercial banks—particularly regional and development-oriented financial institutions operating in emerging economy contexts—utilize fintech-based marketing strategies through sport event sponsorship to simultaneously advance destination branding and inclusive tourism. The study grounds its inquiry in the Uzbekistan and Central Asian context, where rapidly developing digital payment infrastructure intersects with ambitious tourism growth targets and significant financial exclusion among potential visitors. Drawing on four complementary theoretical lenses—Stakeholder Theory, service-dominant logic (S-D logic), the Technology Acceptance Model (TAM), and Sen's Capability Approach—the paper develops an integrative conceptual framework identifying three causal pathways through which fintech innovation, including mobile payment systems, blockchain, artificial intelligence, and alternative lending products, can transform sport event sponsorship into a vehicle for destination competitiveness and social inclusion. The paper also critically engages with the digital divide literature to acknowledge conditions under which fintech sponsorship may deepen rather than reduce exclusion. Seven theoretically grounded propositions are derived, along with four key moderating factors. The study makes conceptual contributions to tourism marketing literature by clarifying the triadic relationship among financial institutions, sport properties, and destination management organizations, and identifies priority directions for empirical research in emerging market contexts.

## 1 Introduction

The global sports tourism market was valued at approximately 587.87 billion USD in 2023 and is projected to grow at an annual rate of 16.1 percent through 2032 (1). This trajectory is being shaped not only by rising consumer appetite for experiential travel, but by a parallel transformation in financial infrastructure: the global fintech industry is expected to generate revenues of 1.5 trillion USD by 2030 (2). The convergence of these two sectors—sports tourism and financial technology—remains undertheorized despite its growing practical significance, particularly in emerging economies where digital financial infrastructure is expanding rapidly while tourism ambitions are high and financial exclusion among prospective visitors remains pervasive. At the same time, the broader topic of tourism–environment–economy relations is equally complex, especially in transition economies where, according to recent findings, there is a significant correlation between tourism growth and CO_2_ emissions in the long run (34).

This paper is motivated by a specific empirical and theoretical gap situated at this intersection. Uzbekistan, the Central Asian country in which the authors are based, offers a paradigmatic illustration of the dynamics this paper seeks to theorize. Over the past decade, Uzbekistan has undergone dramatic liberalization of its tourism and financial sectors. The number of international visitors grew from approximately two million in 2016 to over nine million in 2023, as visa restrictions were eased, heritage sites in Samarkand, Bukhara, and Khiva gained international recognition, and the government adopted tourism as a strategic priority (3, 35). Simultaneously, Uzbekistan's banking sector has pursued fintech-driven modernization, with widespread adoption of digital wallets, QR-code payment networks, and government-backed financial inclusion programs. Yet despite hosting international sport events and sponsoring sporting competitions within the region, no systematic framework exists to understand how banks in this context—or in comparable emerging tourism destinations—can leverage fintech-enabled sponsorship strategies to build destination brands and widen tourism participation. The Central Asian context also usefully highlights the structural constraints that moderate any positive effects: digital infrastructure gaps between urban and rural zones, regulatory frameworks that remain in transition, populations with varying levels of digital financial literacy, and cultural attitudes toward cash that persist alongside rapid digitization.

More broadly, the academic literature on sport sponsorship (4, 5), destination marketing (6), fintech adoption (7), and inclusive tourism (8) has evolved largely in parallel, without a common framework for understanding how these domains interact. This fragmentation is consequential: it makes it difficult for practitioners—whether bank strategists, destination management organization (DMO) directors, or sport event organizers—to design and evaluate strategies that simultaneously address brand competitiveness and social inclusion. It also leaves theorists without a conceptual architecture that captures the mechanisms by which sponsorship activation uniquely enables fintech tools to perform social and marketing work that they could not accomplish outside a high-visibility sporting context.

This paper addresses these gaps by developing an integrative conceptual framework with three specific objectives. First, it theorizes the causal mechanisms by which fintech-based sport event sponsorship shapes destination branding, with special attention to why the sponsorship context is non-redundant. Second, it conceptualizes the pathways through which fintech can support inclusive tourism development, drawing critically on both the positive potential and the documented risks of digital financial inclusion. Third, it identifies the contextual moderating factors—especially those most salient in emerging economy settings—that govern the strength and direction of these effects. The paper is structured as follows. Section 2 provides the theoretical and literature review foundation. Section 3 presents the conceptual framework and seven theoretical propositions. Section 4 discusses implications for theory and practice. Section 5 presents conclusions, limitations, and directions for future research.

## 2 Theoretical background and literature review

This section develops the theoretical and empirical foundations of the integrative framework. It first reviews each of the four theoretical lenses employed, then provides a synthetic integrative subsection that maps their interactions and tensions. It then reviews the substantive literatures on destination branding, sport sponsorship evolution, and inclusive tourism, including a critical engagement with the digital divide and the limits of techno-optimistic accounts of fintech inclusion (Table 1).

**Table 1:** Overview of theoretical perspectives.

Theory	Key authors	Core concepts	Application to framework
Stakeholder theory	Freeman (9); Mitchell et al. (10)	Multi-stakeholder value creation; stakeholder salience (power, legitimacy, urgency)	Maps ecosystem relationships among regional banks, events, DMOs, and tourists in emerging destinations
Service-dominant logic	Vargo & Lusch (11, 12)	Value co-creation; operant resources; service ecosystems	Explains how fintech enables value co-creation in tourism experiences; positions banks as operant resource providers
Technology acceptance model	Davis (13); Venkatesh et al. (14)	Perceived usefulness; perceived ease of use; behavioral intention; trust	Identifies adoption barriers and enablers for tourists encountering fintech in sport event contexts; frames inclusive design requirements
Capability approach	Sen (15); Nussbaum (16)	Human capabilities; functionings; freedoms; social justice	Frames inclusive tourism as capability expansion; evaluates fintech's contribution to real freedoms for marginalized visitor groups
Brand equity theory	Keller (17); Aaker (18)	Brand awareness; associations; perceived quality; loyalty	Conceptualizes destination branding outcomes of fintech-enabled sponsorship activation

Source: Authors’ compilation based on literature review.

Stakeholder Theory, as developed by Freeman (9) and refined by Mitchell et al. (10), asserts that organizational success depends on the effective management of relationships with groups that can influence or be influenced by organizational objectives. Applied to fintech-based sport sponsorship, this theoretical lens reveals a stakeholder ecosystem that extends well beyond the conventional sponsor–property–audience triad. In the context of emerging economies such as Uzbekistan, this ecosystem includes commercial banks, sport event organizers, destination management organizations, national tourism agencies, local governments, fintech infrastructure providers, hospitality firms, and heterogeneous tourist segments differentiated by income, nationality, disability status, and digital literacy.

Mitchell et al.'s (10) salience model, which classifies stakeholder influence according to power, legitimacy, and urgency, is especially useful for understanding why commercial banks in emerging markets are increasingly motivated to develop inclusive sponsorship strategies. As financial inclusion becomes a regulatory and reputational imperative, marginalized tourist segments—the unbanked, those from lower-income backgrounds, visitors from countries without established digital payment networks—acquire greater salience as stakeholders whose needs banks are both obliged and commercially incentivized to address.

Service-dominant logic (S-D logic), as developed by Vargo and Lusch (11, 12), provides a foundational lens for understanding how fintech innovations are transforming sport sponsorship from a primarily transactional arrangement into a platform for value co-creation. S-D logic holds that value is not embedded in products or transmitted unidirectionally from providers to consumers, but is co-created through the integration of resources across a service ecosystem. The core distinction between operand resources (physical assets that must be acted upon) and operant resources (knowledge, skills, and technologies that act upon other resources) is central to understanding the bank's role in fintech-driven sponsorship.

In the fintech-sponsorship-tourism nexus, commercial banks contribute distinctive operant resources: data analytics capabilities, payment infrastructure, financial product design expertise, and established consumer trust. These resources are not simply deployed at an event; rather, they are integrated with the destination's service environment and tourists' own resources—local knowledge, digital devices, cultural familiarity—to co-produce experiences that neither the bank nor the destination could deliver independently. A mobile payment ecosystem deployed in connection with a sport event, for example, generates real-time behavioral data that can be leveraged to co-create personalized destination experiences that extend well beyond the duration of the event itself (19). This co-creative dynamic is what distinguishes fintech-enabled sponsorship from mere brand placement.

The Technology Acceptance Model (TAM), originally formulated by Davis (13) and extended by Venkatesh et al. (14), provides a micro-level behavioral framework for understanding why tourists adopt—or fail to adopt—fintech services in sport event contexts. The original TAM posits that two beliefs drive technology adoption: perceived usefulness (the degree to which a technology improves performance) and perceived ease of use (the degree to which use requires minimal effort). Subsequent extensions of TAM have incorporated additional determinants relevant to fintech adoption, including trust, security perception, financial literacy, and social influence (20–22).

In the context of sport tourism in emerging economies, TAM has particular analytical purchase in identifying adoption barriers for population groups that the framework is intended to include. Research consistently shows that elderly tourists, visitors from low-income countries, and technology-naïve travelers exhibit lower rates of digital payment adoption due to concerns about data security, unfamiliar user interfaces, limited prior exposure to mobile banking, and distrust of digital financial systems (20). These barriers are especially acute in contexts like Central Asia, where smartphone penetration is uneven and digital financial literacy varies sharply across urban and rural populations. Understanding these barriers is essential not merely as a technical design challenge but as a prerequisite for the Capability Approach-grounded argument that fintech sponsorship can expand rather than restrict real freedoms.

The Capability Approach, developed by Sen (15) and extended by Nussbaum (16), provides a normative foundation for evaluating inclusive tourism in terms of whether individuals are able to exercise real freedoms—their capabilities—rather than merely whether formal access exists. Applied to tourism, the approach asks not simply whether fintech payment systems are technically available, but whether all prospective visitors are genuinely capable of using them to pursue tourism participation they have reason to value. This normative reorientation distinguishes the Capability Approach from purely descriptive accounts of fintech adoption and from access-focused framings that treat the provision of technology as equivalent to inclusion.

The World Bank (23, 30) estimates that 1.4 billion adults worldwide remain formally unbanked, making it structurally impossible for them to engage in tourism activities that require credit cards, international transfers, or formal digital payment accounts. Alternative fintech instruments—mobile money, digital wallets linked to national identity systems, buy-now-pay-later products, and microfinancing platforms—can, in principle, expand the functional capabilities of these populations to participate in sport tourism. However, the Capability Approach also cautions against conflating the availability of tools with their transformative use: a mobile payment service is only capability-expanding if potential users possess the digital literacy, internet access, device ownership, and institutional trust required to use it effectively. This normative precision is critical to the inclusive tourism argument developed in the framework below, and it disciplines the propositions so that they specify conditions, not merely intentions.

Destination branding is the process through which destinations develop distinctive identities in the minds of target visitor segments, building brand equity understood through Keller's (17) four dimensions: brand awareness, brand associations, perceived brand quality, and brand loyalty (17, 18, 37). Blain et al. (24, 33) define destination branding as the set of marketing activities that support the creation of a name, symbol, logo, or other graphic that differentiates a destination, consistently conveys the expectation of a memorable travel experience, and consolidates and reinforces the emotional connection between tourists and the destination.

Sport events have been recognized as powerful catalysts for destination brand development (25). They generate media coverage, attract international visitor flows, and create durable associative links between a destination and globally recognized competitions. Fintech sponsorships extend and deepen these effects by creating branded touchpoints that persist across the entire tourism experience—from pre-trip payment planning, through in-venue transactions, to post-trip loyalty engagement—rather than being limited to event signage and broadcast mentions. In emerging economy destinations like those of Central Asia, where destination brands are still being actively constructed rather than merely maintained, fintech-enabled sponsorship creates distinctive modernity associations that can be strategically valuable in competitive international tourism markets.

Sport sponsorship has evolved through four broadly identifiable phases. In the philanthropic era (pre-1970s), sponsorship was principally charitable, with minimal commercial expectations. The commercial era (1970s–2000s) saw sponsorship become a structured marketing tool, with signage, broadcast mentions, and hospitality rights serving as primary value deliverables. The digital and social media era (2000s–2020s) introduced activation (38)—additional investment that brings the sponsorship to life through fan engagement, content creation, and experiential marketing. The fintech-enabled era (2020s onwards) represents a qualitative shift: sponsors are no longer primarily purchasing visibility but are deploying payment ecosystems, data analytics, and inclusive financial products that actively shape how tourists experience destinations (32).

Financial institutions have long been prominent sport sponsors: Visa and Mastercard are official partners of FIFA and the Olympic Games, while numerous regional and national banks sponsor domestic leagues and events. However, the logic of fintech-enabled sponsorship differs from conventional bank sponsorship. Where traditional bank sponsorship achieves exposure and brand association, fintech sponsorship achieves functional integration: the bank's technology becomes structurally embedded in the event and destination experience, generating value for tourists, DMOs, and the bank simultaneously. This is the unique mechanism that the sponsorship context provides and that is absent when fintech tools are deployed in non-sponsorship contexts. Without the high-salience, concentrated visitor population that a sport event provides, the data collection, the co-branding opportunities, and the inclusive finance promotion that the framework proposes would each be significantly less effective.

Inclusive tourism is defined by Scheyvens and Biddulph (8), as tourism that is “welcoming and accessible to all people regardless of their physical ability, income, gender, age, sexuality, ethnicity, or origin.” This encompasses but extends beyond accessible tourism for people with disabilities (26) to address structural financial, cultural, and informational barriers to participation. The inclusive tourism literature emphasizes that equitable access requires active design rather than mere non-discrimination: systems, products, and services must be deliberately constructed to include those who would otherwise be excluded.

A critical strand of this literature—too often absent from optimistic accounts of digital inclusion—is the digital divide scholarship, which documents the ways in which technology-mediated services can reproduce or intensify exclusion rather than reducing it. Several patterns are particularly relevant to the fintech-tourism nexus. First, elderly tourists and those with low digital literacy may find fintech payment interfaces not only unfamiliar but actively alienating, with complex authentication requirements, foreign-language interfaces, and limited error tolerance functioning as exclusionary barriers (22). Second, rural populations in emerging economies frequently lack the stable internet connectivity and smartphone penetration that mobile payment systems presuppose, meaning that infrastructure availability does not translate straightforwardly into capability. Third, algorithmic credit scoring systems that underpin alternative lending products may perpetuate historical biases against certain demographic or national groups, producing outcomes that replicate rather than remedy financial exclusion (27). Fourth, digital payment ecosystems generate vast quantities of behavioral data whose use for personalization and targeting raises legitimate concerns about privacy, consent, and the conversion of tourist experiences into commercial surveillance.

These critical perspectives do not negate the potential for fintech-driven sponsorship to advance inclusive tourism outcomes, but they discipline the analytical framework in important ways. They demand that propositions specify not only the mechanisms through which inclusion can be advanced, but the boundary conditions under which those mechanisms operate and the design requirements that must be met for fintech tools to genuinely expand capabilities rather than merely substituting one form of exclusion for another. This is especially important in the Central Asian context, where digital infrastructure disparities between major cities and rural regions are pronounced, and where national regulatory frameworks governing fintech, data protection, and cross-border payments are still in formative stages.

Financial technology encompasses a broad spectrum of innovations relevant to sport event sponsorship and tourism. Table 2 describes the principal technology categories, their applications in sport tourism contexts, and their implications for inclusive and exclusive outcomes.

**Table 2:** Fintech technologies and their applications in sport tourism.

Technology category	Specific technologies	Sport tourism applications	Inclusive potential	Exclusion risks
Digital payments	Mobile wallets, NFC/contactless, QR codes, wearable payments	Seamless in-venue transactions; integrated destination spending; loyalty accumulation	Reduced cash dependency; multi-currency support for international visitors	Excludes those lacking smartphones or stable connectivity
Blockchain/DLT	NFT ticketing, smart contracts, cryptocurrency payments	Fraud-proof ticketing; programmable experiences; transparent resale markets	Fair ticket distribution; reduced scalping	Technical complexity; requires digital wallets and device ownership
AI/machine learning	Recommendation engines, chatbots, predictive analytics, sentiment analysis	Personalized itineraries; real-time assistance; demand forecasting	Multilingual support; adaptive interfaces for diverse needs	Algorithmic bias; data privacy risks; may reinforce existing preferences
Alternative lending	BNPL, microfinancing, P2P lending, credit scoring algorithms	Flexible payment options for packages; travel financing; budget management	Access for credit-underserved; affordable installments	Algorithmic credit discrimination; risk of over-indebtedness
Open banking/APIs	Account aggregation, payment initiation, data portability	Integrated booking; streamlined verification; unified loyalty across ecosystem	Cross-border interoperability; reduced documentation barriers	Regulatory fragmentation in emerging markets; data sovereignty concerns
RegTech/identity	Digital identity, biometric verification, KYC automation	Frictionless entry; secure transactions; streamlined visa processes	Inclusive identity solutions for underserved groups	Refugees and stateless persons may be excluded; biometric failures

Source: Authors’ synthesis based on Gomber et al. (7), Lee & Shin (27), and critical digital divide literature.

While each of the four theoretical lenses outlined above offers distinct analytical purchase, their genuine contribution to this paper derives from their synthesis rather than their individual application. This section explicitly addresses the interrelationships, complementarities, and productive tensions among the four frameworks, an exercise that prior applications of multi-theoretical frameworks in tourism and sponsorship research have typically neglected.

Stakeholder Theory and S-D logic are mutually reinforcing in this framework. Stakeholder Theory identifies the actors and their power relationships in the fintech-sponsorship-tourism ecosystem; S-D logic specifies the process by which those actors co-create value through the integration of their respective resources. Together, they generate a relational understanding of sponsorship that moves beyond dyadic exchange to capture the systemic character of fintech-enabled destination development. In the Uzbekistan context, this synthesis is particularly apt: the relevant stakeholder network includes not only banks and DMOs but national government agencies whose infrastructure investments underpin whether fintech tools can reach the population segments that the Capability Approach identifies as relevant.

S-D logic and TAM operate at different levels of analysis but are complementary rather than redundant. S-D logic describes the co-creative value logic at the ecosystem level, while TAM specifies the individual-level conditions under which tourists become willing and able participants in that ecosystem. A payment infrastructure that is theoretically value-generating from an S-D logic perspective will fail to produce inclusive outcomes if the tourist-level adoption barriers that TAM foregrounds—perceived complexity, distrust, limited literacy—are not addressed. The two theories thus jointly define both the supply-side conditions (bank capabilities and ecosystem architecture) and the demand-side conditions (tourist acceptance and ability) for the pathways to operate.

The most significant theoretical tension in the framework is between TAM and the Capability Approach. TAM operates within a broadly positivist, behavioral tradition that treats technology adoption as a function of individual attitudes, beliefs, and intentions, with the implicit assumption that if technologies are designed to be usable and useful, adoption will follow. The Capability Approach, by contrast, adopts a structuralist and normative orientation: it insists that the relevant question is not whether individuals choose to adopt technologies, but whether structural conditions make it genuinely possible for them to do so, and whether the outcomes of adoption expand real freedoms. TAM's focus on individual behavioral intention thus risks naturalizing conditions of differential capability—treating as a matter of preference what is in fact a matter of structural constraint. This tension is not merely abstract: in the emerging economy tourism context, a visitor from a rural province who lacks a smartphone, stable internet access, or familiarity with digital payments is not exercising a preference against adoption; they are experiencing a structural capability deprivation. The framework addresses this tension by treating TAM as a tool for understanding adoption dynamics among those who already have the basic material conditions for adoption, while relying on the Capability Approach to evaluate whether those material conditions are equitably distributed and to specify the design requirements for genuinely inclusive fintech sponsorship.

Brand Equity Theory functions in this framework as the domain-specific outcome construct within which the sponsorship and destination marketing literatures are operationalized. It provides the vocabulary for specifying what “improved destination branding” means in measurable terms—awareness, associations, perceived quality, and loyalty—and it links the processes theorized by S-D logic and Stakeholder Theory to outcomes that are observable and evaluable in the destination marketing literature. In this sense, Brand Equity Theory is not an additional theoretical lens so much as the measurement framework through which the branding pathway of the conceptual model can be operationalized in future empirical research.

## 3 Conceptual framework and propositions

Building on the integrated theoretical foundation developed above, this section presents an integrative conceptual model (Table 2) and derives seven theoretical propositions. The model identifies regional commercial banks' fintech capabilities as the core input construct, routes these through sport event sponsorship activation as the necessary enabling mechanism, identifies three causal pathways, specifies four moderating factors, and maps outcomes onto destination brand equity and inclusive tourism. A defining feature of the revised framework is its contextual specificity: it is designed to be applicable to regional and development-oriented commercial banks operating in emerging tourism economies, where the relationship between fintech capability and inclusive outcomes is most consequential and least understood.

A central question that prior conceptualizations of fintech and tourism have left unanswered is why the sport event sponsorship context is necessary rather than merely incidental to the proposed effects. This question deserves an explicit theoretical response, as without it the three constructs—fintech, sponsorship, and destination branding—risk being juxtaposed rather than genuinely integrated.

Sport event sponsorship performs at least three functions that are not replicable through standalone fintech deployment. First, it provides a concentrated, high-salience adoption context. A major sport event brings together a large, bounded population of visitors in a single destination over a defined period. This concentration creates conditions under which fintech tools can be introduced, demonstrated, and normalized at scale in ways that isolated marketing campaigns cannot achieve. Tourists who would not spontaneously adopt a new payment method are more likely to do so when adoption is incentivized, facilitated, and normalized by the event environment—an effect consistent with TAM's social influence construct and well-documented in the diffusion of innovations literature.

Second, sport events generate emotionally salient shared experiences that create uniquely powerful brand association opportunities. The emotional resonance of a memorable sporting moment—a national team's victory, a world record—generates associative encoding in long-term memory that can carry brand associations, including destination brand associations, more effectively than routine commercial exposure. When a bank's payment technology is functionally embedded in this emotionally charged experience, the resulting brand associations benefit from the emotional salience of the event in ways that standard fintech marketing cannot replicate.

Third, sport events create legitimate data collection contexts. In non-sponsorship settings, large-scale collection of tourist behavioral and transactional data raises significant privacy concerns and user resistance. The sport event context provides a bounded, consent-governed framework within which tourists expect their activities to be monitored, data to be collected, and personalized services to be offered. This legitimate data collection opportunity is the foundational input for the engagement amplification pathway, and it is structurally dependent on the sponsorship context.

### 3.1 Pathway 1: barrier reduction through digital payment ecosystems

The first pathway theorizes that fintech-driven payment ecosystems, deployed through sport event sponsorship, reduce the financial and transactional barriers that inhibit tourism participation. Banks sponsoring sport events in emerging economy destinations are increasingly deploying integrated payment ecosystems—mobile wallets, contactless payment terminals, QR-code networks, multi-currency digital wallets, and wearable payment devices—that enable seamless transactions across the full tourism experience, from ticket purchase through accommodation, in-venue spending, and post-trip loyalty redemption.

From a TAM perspective, the perceived usefulness and ease of use of these payment systems positively influence tourist satisfaction and build favorable associations with both the sponsoring bank and the destination. Where transactions are seamless and currency conversion is transparent and low-cost, tourists develop associations of quality, modernity, and tourist-centricity with the destination. From an S-D logic perspective, the digital payment ecosystem functions as a value co-creation platform: banks generate data and build consumer relationships, destinations benefit from increased spending and improved reputation, and tourists receive frictionless service experiences.

Crucially, in the emerging economy context, this pathway also directly addresses financial exclusion barriers. International visitors from developing economies, tourists without credit histories, and visitors accustomed to cash-based economies are disproportionately disadvantaged by payment systems designed for banked, card-carrying visitors from high-income countries. Multi-currency mobile payment support, integration with national digital ID systems, and acceptance of mobile money transfers that bypass formal banking requirements can, if well designed, meaningfully expand the population able to participate in sport tourism at the destination.

**Proposition 1:** Regional commercial banks' deployment of integrated digital payment ecosystems through sport event sponsorship in emerging economy destinations improves destination brand perceptions by enhancing tourists' perceptions of convenience, modernity, service quality, and technological sophistication, provided that digital infrastructure is sufficiently developed and user interfaces are accessible to visitors with varying digital literacy levels.

**Proposition 2:** Fintech payment systems that support mobile money, multi-currency digital wallets, and cash-compatible digital on-ramps, promoted through sport event sponsorship, increase sport tourism participation from underserved visitor segments, including the unbanked, lower-income international tourists, and visitors from developing economies, conditional on the provision of adequate user support and digitally accessible interface design.

### 3.2 Pathway 2a: engagement amplification through AI-driven personalization

The second pathway involves the use of data analytics and artificial intelligence to amplify destination engagement. Sport event sponsorships provide banks with exceptionally rich behavioral data: visitor preferences, movement patterns, spending behaviors, real-time sentiment, and contextual needs. These data, when analyzed through machine learning and predictive modeling (31), enable the construction of personalized destination content and service recommendations that are contextually relevant, temporally appropriate, and individually differentiated.

The mechanism here is distinct from the UGC-amplification mechanism discussed in the following sub-section. AI-driven personalization operates through the bank's own data analytics infrastructure and targets the individual tourist's cognitive and affective engagement with the destination. Personalized AI recommendations—location-aware dining suggestions, transport optimization, accessibility-tailored itineraries for visitors with mobility limitations, multilingual real-time assistance—create the kind of emotionally engaging, contextually relevant, and memorable destination experiences that strengthen brand associations in the manner theorized by Keller (17) and operationalized in S-D logic's notion of superior value propositions.

**Proposition 3:** AI-driven personalization of destination content and services, enabled by fintech data analytics deployed through sport event sponsorship, strengthens destination brand associations and perceived quality among tourists who actively engage with personalized service interfaces, conditional on (a) tourists' willingness to share behavioral data, (b) the cultural and linguistic appropriateness of AI-generated recommendations, and (c) the availability of inclusive AI interfaces for tourists with disabilities or low digital literacy. This proposition is distinctively anchored in the fintech-sponsorship context in that it depends on the rich, multi-dimensional behavioral dataset that only a high-volume sport event sponsorship can generate at sufficient scale and depth.

### 3.3 Pathway 2b: engagement amplification through social Media and UGC

Social media integration operates through a different mechanism that must be analytically distinguished from AI-personalization, with which it shares a pathway label but not a causal logic. Where AI personalization operates through bank-controlled data systems and targets the individual tourist, social media amplification operates through networked peer effects and reaches audiences beyond the event's physical attendees. Fintech-enabled sport event sponsorships that integrate social sharing features, digital collectibles, blockchain-verified experiential tokens, and gamified social engagement mechanisms motivate tourists to generate and share content about their destination experiences.

User-generated content (UGC) amplifies destination brand awareness through several mechanisms: it extends the geographic reach of destination imagery and narrative beyond event attendees; it confers authenticity on destination brand claims through peer endorsement effects (29) that professionally produced content cannot replicate; and it creates persistent digital artifacts of the tourist experience that continue to influence destination perceptions long after the event concludes. The fintech dimension is non-redundant here because the specific tools that motivate UGC creation in this context—blockchain-verified digital souvenirs, shareable payment experiences, gamified loyalty rewards—are distinctive to the fintech-sponsorship context and would not be available through conventional sponsorship activation.

**Proposition 4:** Integration of social media sharing features and blockchain-verified digital experiential artifacts within fintech-powered sport event sponsorships generates user-generated content that amplifies destination brand awareness and perceived authenticity among audiences beyond event attendees, conditional on (a) tourists' digital connectivity and social media engagement, and (b) the design of fintech tools that provide genuine sharing incentives beyond generic loyalty points. This proposition is grounded in the fintech-sponsorship context in that the specific UGC motivators it describes—digital tokens, verifiable experiential records, gamified fintech rewards—are uniquely available through fintech-enabled sponsorship activation.

### 3.4 Pathway 3: financial democratization through inclusive financial products

The third pathway addresses how fintech innovations enable banks to extend inclusive financial products that can widen sport tourism participation among economically marginalized groups. Transaction costs, currency exchange friction, credit restrictions, and documentation requirements have historically limited sport tourism participation among the financially disadvantaged, visitors from developing economies, and those without conventional banking relationships (28). From the Capability Approach perspective, these barriers constitute capability deprivations: they prevent individuals from engaging in tourism they have reason to value, not as a result of preferences but as a result of structural constraints.

Fintech products including buy-now-pay-later (BNPL) options for tour packages, microfinancing for sport tourism experiences, peer-to-peer lending platforms for travel funding, and alternative credit scoring that incorporates non-traditional financial signals can address these constraints by extending purchasing power to credit-underserved populations. However, as the digital divide literature cautions, these products carry their own exclusion risks: BNPL products can create debt traps for financially vulnerable consumers; algorithmic credit scoring may replicate historical biases against certain demographic groups; and microfinance products may require digital literacy and device ownership that not all intended beneficiaries possess. The Capability Approach demands that inclusive financial products be designed to genuinely expand real freedoms rather than to substitute formal inclusion for substantive capability.

When promoted through high-visibility sport event sponsorships, these inclusive products can reach previously unaddressed market segments and position the sponsoring bank as a socially responsive institution, generating brand equity benefits among value-conscious consumer segments—particularly millennials and Gen Z travelers, for whom corporate social responsibility is a demonstrated factor in brand evaluation.

**Proposition 5:** Inclusive fintech products, including microfinancing, BNPL options with transparent cost structures, and alternative credit products with non-discriminatory scoring algorithms, promoted through sport event sponsorships in emerging economy destinations, increase sport tourism participation among economically disadvantaged visitor groups, conditional on the products' accessibility, affordability, and design compliance with financial protection standards for vulnerable consumers.

**Proposition 6:** Regional banks that credibly promote inclusive fintech products through sport event sponsorships in emerging economy destinations develop differentiated brand identities among socially conscious tourist segments, conditional on the demonstrable impact of the inclusive products—that is, conditional on the bank's actual rather than merely rhetorical commitment to financial democratization. Reputational benefits are contingent on consumer trust, which requires transparent impact reporting and consumer protection practices.

The strength and direction of all three pathways are moderated by four contextual factors that are particularly salient in emerging economy settings. These moderators are derived from contingency theory and institutional theory and reflect the conditions that determine whether fintech-driven sponsorship produces positive destination branding and inclusive tourism outcomes.

The first moderating factor is digital infrastructure maturity, measured by internet penetration rates, smartphone ownership, digital payment terminal coverage, and network reliability. In Uzbekistan and comparable Central Asian destinations, this factor exhibits significant urban-rural variation, with major cities possessing infrastructure broadly comparable to middle-income regional norms, while rural and remote areas face significant connectivity gaps. This unevenness means that inclusive strategies designed for urban event attendees may be structurally inaccessible to domestic rural visitors.

The second is the regulatory environment, including fintech licensing frameworks, data protection legislation, cross-border payment facilitation, and consumer protection provisions for alternative lending products. Emerging economies in transition, including Uzbekistan, have been actively developing fintech regulatory frameworks, but gaps between regulatory intent and implementation capacity mean that the enabling conditions for some fintech instruments are more fragile than in established regulatory environments.

The third moderating factor is cultural receptivity, encompassing attitudes toward digital financial services, trust in electronic payments, the persistence of cash culture, and group-level norms around technology adoption and data sharing. In societies with historically strong cash cultures and limited institutional trust in formal financial systems—patterns found in several Central Asian contexts—the adoption of fintech payment systems may require significant trust-building investment before the pathways described above can operate effectively.

The fourth factor is stakeholder collaboration intensity—the degree to which banks, DMOs, event organizers, government agencies, and technology providers align incentives and coordinate activities. In emerging economy destinations where institutional coordination mechanisms are less developed than in established tourism markets, the value co-creation logic of S-D logic may be weakened by fragmented stakeholder relationships and misaligned objectives.

**Proposition 7:** The positive effects of fintech-driven sport event sponsorships on destination brand equity and inclusive tourism outcomes in emerging economy settings are contingent upon: (a) digital infrastructure maturity sufficient to ensure broad-based access to fintech tools, (b) a supportive regulatory environment that enables fintech deployment and protects consumers, (c) a cultural receptivity to digital financial services that supports adoption without coercive digital substitution, and (d) high-intensity stakeholder collaboration among banks, DMOs, event organizers, and government bodies. The direction of effects may be negative in contexts where any of these moderating conditions is severely deficient (Table 3).

**Table 3:** Summary of theoretical propositions.

Prop.	Pathway	Core statement	Theoretical foundation	Expected outcomes
P1	Barrier reduction	Digital payment ecosystems enhance destination brand perceptions through convenience and modernity (conditional on infrastructure adequacy and interface accessibility)	TAM; S-D Logic; Brand Equity	↑ Destination image; ↑ Perceived quality
P2	Barrier reduction	Diverse, inclusive payment options increase tourism participation from excluded segments (conditional on accessible design and user support)	Capability Approach; Stakeholder Theory; TAM	↑ Participation rates; ↑ Market inclusion
P3	Engagement amplification (ai personalization)	AI-driven personalization strengthens destination brand associations (conditional on data sharing, cultural fit, and inclusive interface design; distinctively enabled by event-scale data)	Brand Equity; S-D Logic; TAM	↑ Brand associations; ↑ Perceived quality; ↑ Memorability
P4	Engagement amplification (UGC/social media)	Fintech-specific digital artifacts and sharing incentives generate UGC that amplifies destination awareness beyond event attendees (conditional on connectivity and genuine sharing incentives)	Network Effects; UGC Theory; S-D Logic	↑ Awareness; ↑ Reach; ↑ Perceived authenticity
P5	Financial democratization	Inclusive fintech products increase participation among disadvantaged groups (conditional on product accessibility, affordability, and consumer protection compliance)	Capability Approach; Financial Inclusion Theory	↑ Real capability; ↑ Participation rates
P6	Financial democratization	Credible inclusive initiatives enhance brand equity among conscious consumers (conditional on demonstrable impact and consumer protection practices)	CSR Theory; Brand Equity; Capability Approach	↑ Brand loyalty; ↑ Differentiation
P7	Moderating	Effects are contingent on digital infrastructure maturity, regulatory environment, cultural receptivity, and stakeholder collaboration intensity; direction of effects may be negative where conditions are severely deficient	Contingency Theory; Institutional Theory; Capability Approach	Conditional effects; context-dependency; potential reversal under adverse conditions

Source: Authors’ elaboration.

## 4 Discussion and implications

This paper makes several interconnected theoretical contributions that collectively advance the understanding of fintech, sport sponsorship, and tourism as a coherent domain of inquiry rather than a coincidental intersection of separate literatures.

First, the paper extends Stakeholder Theory by formalizing the multi-actor ecosystem through which fintech-enabled sport sponsorships create value in emerging economy tourism destinations. Prior stakeholder analyses of sponsorship have concentrated on the triad of sponsor, property, and audience. The present framework extends this to a network that includes regional development banks, DMOs, government agencies, technology infrastructure providers, and heterogeneous tourist segments distinguished by digital capability, income, and mobility. This network-level stakeholder conceptualization is consistent with Cornwell's (5) call for systemic sponsorship research and addresses the observation that conventional sponsorship models inadequately capture the relational complexity of fintech-enabled arrangements.

Second, the framework extends S-D logic's account of value co-creation in tourism by specifying fintech innovations as categorically distinct operant resources that transform the sponsorship relationship from a visibility exchange into a value co-creation architecture. The distinction between AI personalization and UGC amplification as distinct sub-mechanisms within the engagement amplification pathway reflects S-D logic's emphasis on the heterogeneity of resource integration processes and corrects a conflation present in earlier iterations of the framework. Importantly, the co-creative dynamic the framework describes is causally dependent on the sponsorship context: the scale, emotional salience, and data richness of a sport event are what make fintech-driven co-creation practically achievable rather than theoretically possible.

Third, the paper makes a substantive contribution to inclusive tourism theory by providing the first systematic application of the Capability Approach to the question of how fintech-enabled sponsorship can expand or restrict real freedoms for marginalized visitor groups. This contribution goes beyond prior access-focused analyses by specifying the conditions under which fintech tools genuinely constitute capability expansion vs. those under which they reproduce exclusion in a digital register. The integration of digital divide critiques into the capability analysis—and the derivation of boundary conditions in Propositions 2, 3, 5, and 6 that specify design requirements for genuine inclusion—represents a normative precision that the existing inclusive tourism literature has not previously achieved in relation to digital financial technologies.

Fourth, the paper clarifies the conceptual relationship between fintech sponsorship and destination brand equity by theorizing fintech touchpoints as persistent brand association generators that operate across the pre-trip, event, and post-trip experience rather than being confined to the event itself. This temporal extension of the sponsorship–branding nexus is particularly important for emerging economy destinations, where destination brand equity is not yet established and where the modernity and accessibility associations created by fintech sponsorship can contribute distinctively to international competitive positioning.

Fifth, the paper makes a methodological contribution to conceptual framework development in tourism research by demonstrating how multi-theoretical frameworks can be integrated synthetically rather than additively. The integrative subsection in the literature review—which maps the complementarities between Stakeholder Theory and S-D logic, the multilevel complementarity between S-D logic and TAM, the normative tension between TAM and the Capability Approach, and the outcome-specification role of Brand Equity Theory—provides a model for theoretically coherent multi-lens frameworks that generate more precise propositions than frameworks that apply each theory to a separate analytical compartment (Table 4).

**Table 4:** Stakeholder-specific implications and action areas.

Stakeholder	Key implications	Recommended actions	Emerging economy priorities
Commercial banks	Sport sponsorship as fintech showcase and financial inclusion platform; dual brand-social impact objectives	Integrate accessible payment ecosystems; develop inclusive financial products with consumer protections; leverage data for personalization; measure and report social impact	Design for digital literacy heterogeneity; invest in offline-capable payment fallbacks; partner with national financial inclusion programs
DMOs	Strategic fintech partnerships enhance destination competitiveness and accessibility	Seek fintech sponsor partnerships; invest in digital infrastructure; enable consented data sharing; co-create inclusive visitor experiences	Align sponsorship strategies with national digital inclusion and tourism development agendas; address urban-rural infrastructure gaps
Event organizers	Enhanced sponsorship value through fintech integration opportunities	Create fintech-enabled sponsorship packages; facilitate technology integration; provide behavioral data access; enable activation zones	Ensure fintech activation zones are accessible to visitors with disabilities and low digital literacy; provide multilingual support
Policymakers	Regulatory environment critical for fintech-tourism synergies and inclusive outcomes	Enable cross-border payments; balance data protection with personalization; support financial inclusion; invest in digital infrastructure	Develop adaptive fintech licensing frameworks; protect financially vulnerable consumers from predatory alternative lending products; invest in rural digital infrastructure
Tourists	Enhanced experiences, reduced barriers, greater accessibility and affordability—but contingent on design quality and infrastructure	Engage with fintech solutions that are clearly beneficial; provide feedback on accessibility barriers; support inclusive design advocacy	Access consumer protection resources; be aware of data sharing implications; use inclusive fintech products that expand rather than restrict financial freedom

Source: Authors’ elaboration based on framework implications.

For regional commercial banks in emerging economies, the framework's practical message is that sport event sponsorships offer a uniquely powerful context for demonstrating fintech capabilities to concentrated visitor populations while simultaneously addressing financial inclusion objectives. Banks in Uzbekistan and comparable destinations that are active sponsors of regional sport events can use these sponsorships to normalize digital payment adoption, distribute inclusive financial products, and generate the data assets needed to personalize destination services—provided they invest in designs that address the barriers identified by TAM and the Capability Approach. Sponsorships that are oriented exclusively toward brand visibility, without integrating inclusive product access and digitally accessible interface design, are likely to amplify existing exclusion patterns rather than remediate them.

For destination management organizations in emerging tourism economies, the framework suggests that fintech sponsor partnerships represent a qualitatively different category of value relative to conventional sponsorship, because they generate persistent digital infrastructure for the destination rather than merely temporary visibility uplift. DMOs should prioritize partnerships with banks that can demonstrate genuine inclusive product portfolios and that commit to post-event legacy infrastructure—maintaining the payment ecosystems, loyalty networks, and data-sharing arrangements that continue to generate visitor value after the sport event concludes (36).

For policymakers, particularly those in Central Asia and comparable emerging economy tourism markets, the framework highlights the regulatory environment's critical role as a moderator. The positive effects theorized in Propositions 1 through 6 are conditional on regulatory frameworks that enable fintech deployment, protect consumers from predatory products, and ensure that data generated by fintech-enabled sponsorships cannot be used in ways that violate visitor privacy or enable discriminatory targeting. Adaptive fintech regulatory frameworks that create space for innovation while maintaining consumer protection standards are essential infrastructure for the inclusive tourism outcomes the framework describes.

**Figure 1:**
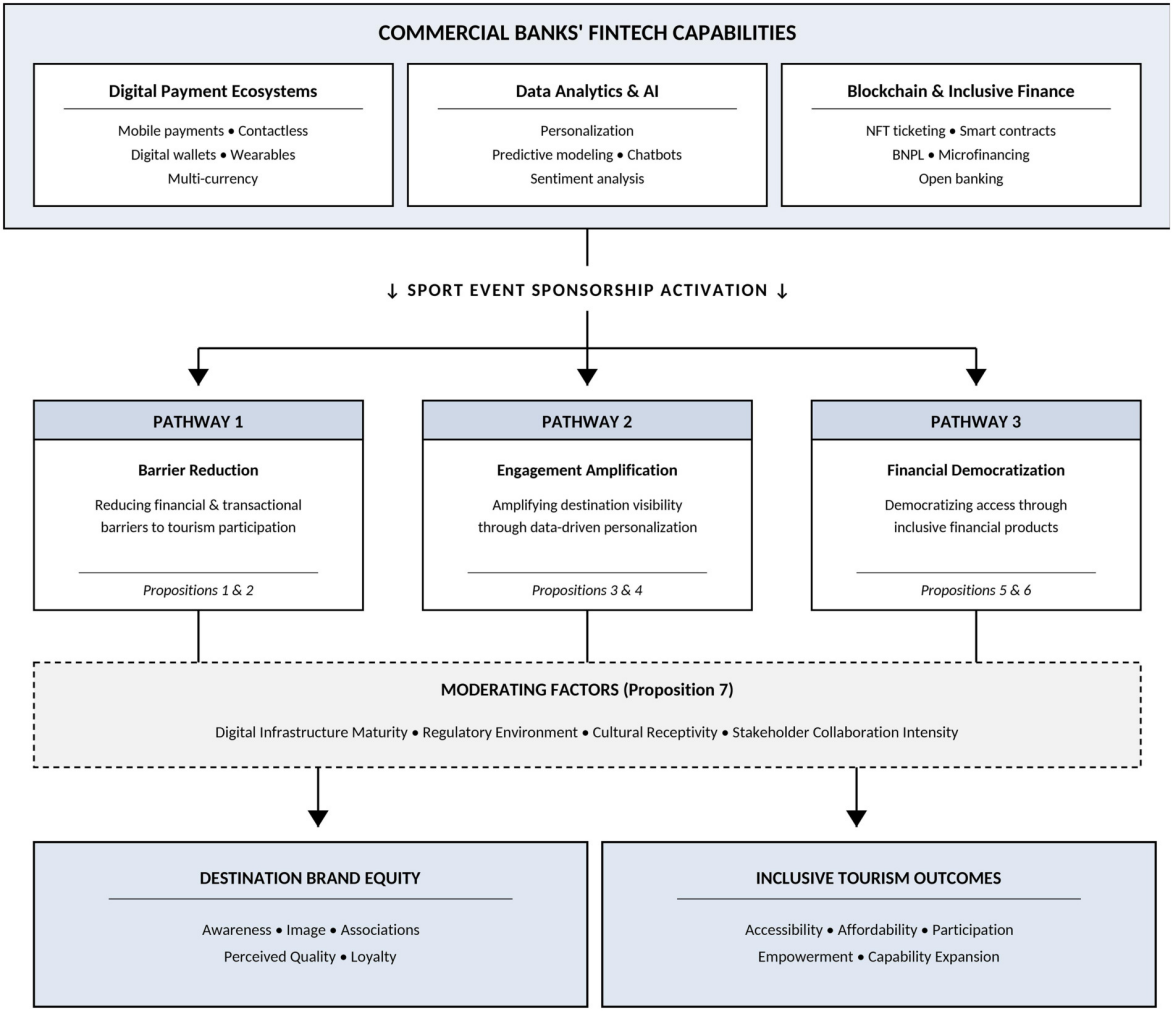


## 5 Conclusions

This paper has developed an integrative conceptual framework theorizing how regional commercial banks' fintech capabilities, deployed through sport event sponsorship, can advance destination brand equity and inclusive tourism outcomes in emerging economy settings. The framework integrates four theoretical lenses—Stakeholder Theory, service-dominant logic, the Technology Acceptance Model, and the Capability Approach—in a synthetic rather than additive manner, identifies three causal pathways operating through distinct mechanisms, and derives seven theoretically grounded propositions with explicit boundary conditions. Critically, the framework specifies why the sport event sponsorship context is non-redundant: the concentration of visitors, the emotional salience of sporting experience, and the legitimate data collection context that sport events provide are each conditions that enable fintech tools to produce destination branding and inclusive tourism outcomes that standalone fintech deployment cannot replicate. The framework is grounded in the Central Asian and Uzbekistan context but is designed to be applicable to other emerging economy tourism destinations sharing comparable structural conditions.

Several limitations of this conceptual paper should be acknowledged. The propositions require empirical validation across diverse geographic, cultural, and institutional contexts, and the framework's predictions may require modification in response to empirical findings. The treatment of commercial banks, while refined by the focus on regional and development-oriented institutions in emerging economies, still encompasses a heterogeneous population of organizations whose specific capabilities and constraints will vary substantially. The rapidly evolving fintech landscape, including emerging technologies in central bank digital currencies, DeFi, and the metaverse, may generate new sponsorship mechanisms not captured in the current framework. Finally, while the paper critically engages with the digital divide and acknowledges the conditions under which fintech sponsorship may deepen exclusion, it does not fully operationalize how these negative dynamics can be detected and mitigated in practice—a gap that future research should prioritize.

These limitations define several productive directions for future research. Most urgently, the propositions should be subjected to empirical testing using quantitative designs—structural equation modelling of survey data among sport tourists and local visitor populations, or quasi-experimental evaluation of fintech-enabled vs. conventional sponsorship activations at comparable events—in contexts where the moderating conditions vary, to test the conditional logic of Proposition 7. Qualitative case study research examining specific triadic relationships between banks, sport events, and destinations in Uzbekistan and comparable Central Asian settings would provide the contextual depth that survey research cannot achieve. Longitudinal research designs are needed to assess whether the brand equity effects and capability expansion outcomes theorized in the propositions persist beyond the event period. Comparative research across destinations with different levels of digital infrastructure, financial inclusion, and cultural receptivity would allow systematic testing of the moderating relationships. Finally, research explicitly addressing the negative side of fintech-based sponsorship—digital exclusion dynamics, data privacy violations, algorithmic discrimination, and the conditions under which BNPL and microfinance products create rather than reduce financial vulnerability—would provide the balanced empirical foundation needed to develop genuinely inclusive fintech sponsorship practice.
